# Sexually dimorphic activation of dopaminergic areas depends on affiliation during courtship and pair formation

**DOI:** 10.3389/fnbeh.2014.00210

**Published:** 2014-06-11

**Authors:** Mai Iwasaki, Thomas M. Poulsen, Kotaro Oka, Neal A. Hessler

**Affiliations:** ^1^RIKEN Brain Science InstituteWako-shi, Japan; ^2^Department of Biology, Keio UniversityYokohama, Japan

**Keywords:** social behavior, courtship, video tracking, dopamine

## Abstract

For many species, dyadic interaction during courtship and pair bonding engage intense emotional states that control approach or avoidance behavior. Previous studies have shown that one component of a common social brain network (SBN), dopaminergic areas, are highly engaged during male songbird courtship of females. We tested whether the level of activity in dopaminergic systems of both females and males during courtship is related to their level of affiliation. In order to objectively quantify affiliative behaviors, we developed a system for tracking the position of both birds during free interaction sessions. During a third successive daily interaction session, there was a range of levels of affiliation among bird pairs, as quantified by several position and movement parameters. Because both positive and negative social interactions were present, we chose to characterize affiliation strength by pair valence. As a potential neural system involved in regulating pair valence, the level of activity of the dopaminergic group A11 (within the central gray) was selectively reduced in females of positive valence pairs. Further, activation of non-dopaminergic neurons in VTA was negatively related to valence, with this relationship strongest in ventral VTA of females. Together, these results suggest that inhibition of fear or avoidance networks may be associated with development of close affiliation, and highlight the importance of negative as well as positive emotional states in the process of courtship, and in development of long-lasting social bonds.

## Introduction

Successful dyadic interactions with individuals of the opposite sex are critical for reproductive success. In many species, both short and long-term processes of courtship and pair bonding engage intense emotional states which control approach or avoidance behavior. Among the best characterized forms of courtship is that of male songbirds singing to attract a preferred female. Courtship in some species, such as zebra finches, is a dyadic interaction in close proximity. While a male sings, the target female listens and decides whether to continue courtship interaction based on her judgment of the song and other factors. As a final step, the female may produce a copulation solicitation display (CSD), involving a horizontal posture and tail quivering, which the male can view (Morris, 1954). Thus, during the courtship interaction, both individuals make ongoing judgments regarding the other, and produce behavioral signals reflecting these decisions. In addition, it is likely that brain activity reflecting the choice of each individual can be influenced by responses of the other. For example, the relative activation of brain reward networks in a male attracted to a female bird should depend on whether she makes a positive or negative response to his singing.

Courtship behavior of vertebrates is controlled by largely evolutionarily common areas known as the social brain network (SBN:Newman, 1999; Goodson, 2005). As emphasized in a recent review, reward and motivation related dopaminergic areas are an essential component of the SBN (O'Connell and Hofmann, 2011). These areas are involved in motivational processes for affiliative behavior and other natural rewards (Kelley and Berridge, 2002; Stuber et al., 2008). Further, a critical role of dopaminergic systems in courtship and pair formation has been demonstrated in mammals (Aragona et al., 2006) and suggested in birds (Goodson et al., 2009; Alger et al., 2011; Pawlisch et al., 2012; Banerjee et al., 2013; Iyilikci et al., 2014). In songbirds, both dopaminergic and non-dopaminergic neurons in VTA are selectively active when male zebra finches sing to court females but not when they sing in a non-courtship context (Yanagihara and Hessler, 2006; Hara et al., 2007; Huang and Hessler, 2008). Singing of males may be interpreted as a sign of attraction to the female, and the level of VTA modulation is high when males sing to females but lower when males only see the female but do not sing (Yanagihara and Hessler, 2006). Thus, activity in VTA may reflect the degree of motivation of a male to mate with a female, based on sensory characteristics of the female and a male's current internal state.

Previous studies in songbirds have focused almost exclusively on males, because of their prominent courtship display. However, as noted above, courtship requires a critical decision by females—whether to accept the singing male's proposal. Further, most studies have used reduced experimental paradigms, in which females and males can't physically interact. For example, males and females are often kept in separate cages during courtship experiences, so that both birds are aware at some level that there was no access to the other (Sasaki et al., 2006; Yanagihara and Hessler, 2006; Hara et al., 2007; Huang and Hessler, 2008). It seems likely that brain networks involved in affiliative decision making may be fully engaged only when normal outcomes of interaction are possible. Therefore, in order to investigate brain activation related to courtship and pairing, we wished to examine behavioral interactions in a more natural setting, so that both birds have the opportunity to complete behavioral approach or withdrawal behaviors. Also, as the majority of previous studies examined brain function in only male birds, we examined both sexes. We tested whether activation of previously characterized areas related to male courtship, as well as other dopaminergic areas associated with the SBN, is characteristic for pairs that have behaviorally defined negative or positive experience related to courtship and pairing.

In order to quantify behavioral features of interaction, we developed a system for automatically tracking the position and movement of both birds. This allowed us to systematically quantify specific measures related to affiliative behavior, such as inter-bird distances. During a third daily interaction session, there was a range of affiliation level among pairs of birds. As expected, among these randomly selected pairs of birds some displayed mainly positive social behaviors and some mainly negative. Thus, we chose to characterize relative affiliation of pairs by a valence score ranging from −1 to +1. The valence during the third session was strongly predicted by a specific female-male interaction during their initial meeting session: pairs in which aggressive behavior occurred during male courtship singing developed negative valence, while those lacking aggression during courtship had positive or neutral valence.

The level of activation of the immediate early gene (IEG) protein c-Fos (product of c-fos) following this final interaction session was quantified in several dopaminergic areas included in the SBN. As a potential neural system involved in affiliation, the level of c-Fos in putative dopaminergic neurons in A11/PAG was selectively reduced in positive valence pairs for females, but not males. Further, activation of non-dopaminergic neurons in VTA was negatively related to pair valence, with this relationship strongest in ventral compared to dorsal VTA of females. Together, these results suggest that inhibition of fear or avoidance networks may be associated with development of a female's close affiliation with a male. The lack of such reduction in males could indicate a slower development of affiliation, or a slower recognition of ongoing affiliative behavior of the female partner. These results highlight the importance of both positive and negative emotional states in the process of courtship, and in development of long-lasting social bonds.

## Materials and methods

### Animals and behavior protocol

Adult zebra finches (19 each female and male) bred in our laboratory facility were used in this study. All birds lived in a cage with parents and siblings until 70–100 days old, and thereafter in a communal cage with others of the same sex. Birds were adapted to living in a small cage (27 × 27 × 20 cm) containing two perches inside a sound attenuation box for several days to weeks before experimental observations began. To eliminate disruptive effects of social isolation on dopaminergic systems (Jones et al., 1990; Huang and Hessler, 2008; Feder et al., 2009) birds were housed during this period with a zebra finch of the same sex.

Following this, one female and one male from each cage were transferred to a separate identical cage for 30 min, over three successive days. Each day, this interaction began 3 h after the light period began, to minimize nonspecific arousal related activation of dopaminergic neurons (Lu et al., 2006; Takahashi et al., 2010), as well as interference with social motivation by strong thirst and hunger upon waking. Both birds were introduced into a neutral cage for interaction sessions, to reduce potential dominance effects caused by cage residence. In one pair, an unfamiliar female was placed in the cage during the third session with a male who had been paired with another female during the two previous sessions. Behavioral and neural results from this third session only were combined with other results. All procedures were reviewed and approved by the RIKEN Animal Experiments Committee (Approval ID: H23-1-223).

### Anatomical analysis

One hour after the third interaction session began (both birds remained in the cage for the 30 min after interaction session), both birds were quickly anesthetized (Sodium pentobarbitol, IM) and perfused with 0.1 M PBS/0.4% heparin followed by 4% paraformaldehyde to fix brain tissue. Brains were removed from the skull, postfixed for 2 days, soaked in 0.1 M PB/30% sucrose and embedded in gelatin. Frozen brains were cut in 40 μm sections with a cryostat, collected in cryoprotectant, and stored at −30°C. The immunohistochemical procedure began with inactivation of endogenous peroxidase by 2 min in 0.3% H_2_O_2_, 30 min in blocking solution (1% tritonX + 5% normal goat serum in PBS), and overnight incubation in rabbit anti-c-Fos antibody (Santa Cruz Biotechnology, Santa Cruz, California, USA) diluted 1:5000 in 0.5% tritonX + 2.5% normal goat serum/PBS. Sections were incubated for 1 h in anti-rabbit IgG (H+L) biotinylated goat antibody (Vector Laboratories, Burlingame California, USA) diluted 1:500 in 0.5% tritonX + 10% normal goat serum/PBS, 30 min in VECTASTAIN Elite ABC Standard Kit (Vector Laboratories, Burlingame California, USA), and antibody was visualized by incubation in DAB substrate kit for peroxidase (Vector Laboratories, Burlingame California, USA). TH was labeled by overnight incubation in mouse monoclonal anti-rat tyrosine hydroxylase antibody (Acris Antibodies, Herford, Germany) diluted 1:1000 in 0.5% tritonX + 2.5% normal goat serum/PBS, 30 min in VECTASTAIN Elite ABC Standard Kit, 13-min reaction of TMB substrate kit for peroxidase (Vector Laboratories, Santa Cruz California, USA), and decoloration of TMB until DAB stain became visible. Sections were rinsed three times in 0.1 M PBS between each step.

The number of neurons containing label for both TH (blue, cytosol) and c-Fos (brown, nucleus), and for TH only were counted by direct observation with a 20× objective (OLYMPUS UPlanFl 20×/0.50, 8/0.17, Tokyo, Japan) in dopaminergic groups (from rostral to caudal) A14, A15, A11 in stratum cellulare internum (SCI), A10 in caudal VTA, and A11 in CG. In VTA, the number of neurons containing label for c-Fos only were counted using a grid to divide the region 300–550 μm from midline into dorsal and ventral subregions.

### Video tracking

**Figure 1:**
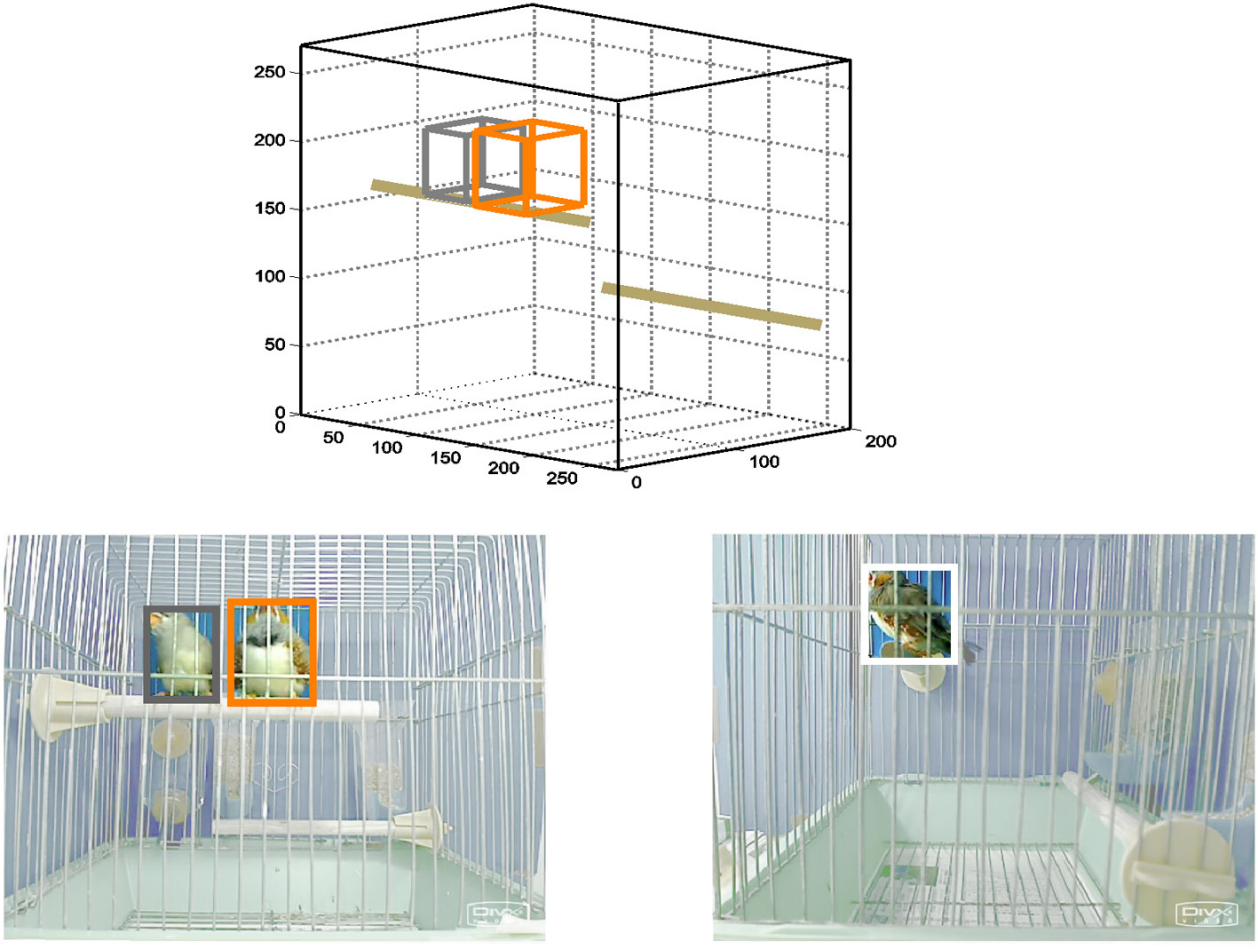
**Schematic of system for automatic tracking of individual birds during interaction sessions.** Lower left and right panels are images obtained by two webcams positioned outside the front and side of a cage containing a female and a male zebra finch. Software could identify and discriminate between female and male birds (outlined in gray and orange, respectively) based on color differences. In the side view, detection of female bird is prevented by the obscuring male. Positions detected by both cameras were combined to obtain three-dimensional coordinates of both birds in the cage (upper panel, axis labels indicate dimensions in mm).

Paired videos (30 fps, VGA resolution) were recorded by two Logitech web cameras (Logitech International S.A., Switzerland) positioned at perpendicular points outside of the cage (front and side, Figure 1). Videos were processed using Matlab (MathWorks, Natick, Massachusetts, USA) to detect the outline of both birds, and these boundaries were combined mathematically to identify for each video frame the three-dimensional location of the female and male within the cage. This procedure required: 1. determination of the background image of cage apparatus not including birds, 2. subtraction of each frame of video from background to detect birds, 3. identification of female and male birds based on characteristic feather colors, 4. combination of views from both cameras to calculate three-dimensional position of both birds.

As most behavior relevant for this study occurs during perching, an example of the system's performance during a perch event shows a critical advantage allowed by stereo tracking (Figure 1). Positions of both a female (gray) and a male (orange) calculated using both views were clearly distinct from the information extracted from the side-view camera alone. The system was able to accurately identify both birds in almost all video frames (median = 94% of frames), but sometimes could not separately distinguish the female and male when they were in contact with each other (median = 1% of identified frames), or when characteristic features became obscured (remainder of missing frames).

Here, we intended to compare neural activation in pairs who experienced relatively positive or negative courtship interaction. When unfamiliar birds first meet, their interaction likely entails a high level of general arousal. In an attempt to increase the relative importance of specific judgment regarding the other bird, we assessed their behavior during a third successive interaction session, when they have had some experience with each other. Based on previous behavioral studies, we expected that some pairs would at this point have began initial stages of pair bonding, and some would have not (Caryl, 1976; Silcox and Evans, 1982).

Position information was used to generate an estimate of the strength of affiliation for each pair. We chose relatively simple static and dynamic features that included information about inter-bird interactions, and could be reliably calculated (were not sensitive to small changes in measurement windows). Three stationary position measures used were: % of total time both birds spent sitting on the same perch (X2), % of total time both birds spent sitting on opposite perches (X5), and average distance between birds when they both sat on the same perch for longer than 100 video frames (3.33 s, X3). Two movement related measures used were: the number of times either bird left a perch that both were sitting on for over 100 frames (X1), and the average maximum distance between both birds during these movements from the perch, within 3.33 s (X4). This measure quantifies how far the actively “withdrawing” bird moves from the other. In order to relate these measures to pair affiliation status, a principal component analysis (PCA) was performed on them (princomp, Matlab). Based on the two major components, a single scalar score was calculated as PC1^*^0.25 + PC2. As we expected and observed both positive and negative social interactions that varied between pairs, we normalized this scalar score over the range of −1.0 to +1.0, as a measure of pair valence.

Singing periods were detected based on the acoustic power of the video audio channel (Matlab). All singing was confirmed by visual inspection to be of the “directed” type used during courtship, with males oriented toward females while performing stereotyped dance movements. Singing bouts, used for examining female and male behavior associated with courtship, were defined as successive songs that had less than 3 s long pauses between them.

During such bouts of male courtship singing, copulation, and aggressive behavior were detected by scanning for video frames where both birds were adjacent or body centers were within 7 cm. Successful copulations were counted as those in which the male remained above the female for longer than 1 s, while both birds remained relatively stationary. Aggressive behaviors during courtship were detected by scanning video for rapid beak pecking of the female or male targeting the other bird. For each such aggressive event, the pecked bird responded by either retreating (fleeing) or not retreating (remaining in the same position). From these data, we obtained for each interaction session the number of aggressive behaviors by either bird toward the other, and the response of the target bird to each aggression.

Comparisons between groups were made with parametric or nonparametric statistical tests based on results of Lilliefors goodness of fit tests for normality.

## Results

### Video tracking and valence quantification

The positions of female and male birds interacting in the same cage were automatically tracked by a simple video system. By combining orthogonal views (Figure 1, front and side), this system calculated the three-dimensional position of both birds in each frame of a video (female, gray box; male, orange box). The use of such a system was critical for our experiments, as normal bird behavior utilizes three-dimensional space, and thus occasionally a view of one camera could be obscured (Figure 1 lower right).

Results of tracking were used to quantify several measures related to both static and dynamic positions of individuals, as well as behavioral interactions. The distance in space between two individuals is negatively related to their affiliative activity. In the cages we used, one feature relevant to this is the absolute inter-bird distance during epochs when both birds were sitting on the same perch (X3). Because the cage contained two perches, the relative amount of time both birds spent on the same perch also seemed related to affiliation (X2). Besides these features which provide information of positive interaction within pairs, several features containing information about negative or neutral behaviors were: time spent sitting on opposite perches (X5), the relative level of jumping between perches (X1), and the maximum distance between birds during such perch jumping episodes (X4, see Materials and Methods for details of quantification).

**Figure 2:**
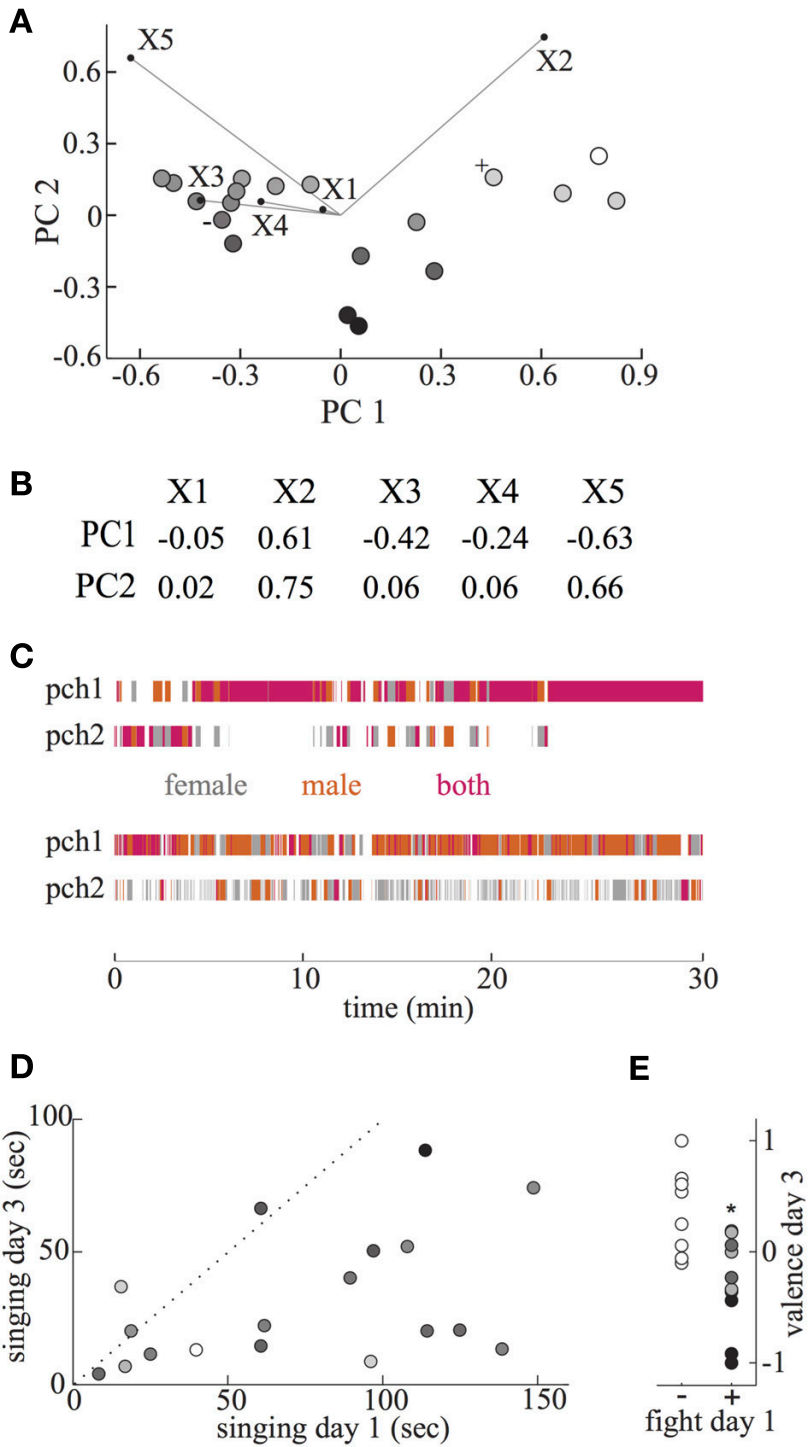
**Information from tracking system used to obtain pair valence, and relation of courtship behaviors to valence. (A)** A Principal Component Analysis (PCA) was performed on five behavioral features of interactions of female and male birds. Results from the first two principal components, which accounted for 88% of the variance, are plotted for each pair. Mappings of each behavioral factor are overlaid projected within this plane, with the length of the line equal to the PC1 and PC2 components. The five behavioral features are: X1—% time spent jumping between perches, X2—% time both birds on same perch, X3—average distance between birds when both on same perch, X4—distance moved when jumping between perches, X5—% time both birds on opposite perches. Symbols for pairs which had positive and negative valences, illustrated in **(B)**, are labeled with “+” and “−,” respectively. The final calculated valence of each pair is coded with a grayscale map, from black = −1 to white = +1. B. Relationship of PC1 and PC2 to the five behavioral features used. **(C)** Presence of female and male birds on either perch in the cage for each video frame. Video frames when only the female or male was on perch 1 (pch1) or perch 2 (pch2) are indicated with gray and orange, respectively. Frames when both female and male were on the same perch are indicated by magenta. Upper and lower perch position plots represent data from day 3 recordings of positive valence (0.38, indicated by “+” to left of symbol in **A**) and negative valence (−0.24, indicated by “−” to left of symbol in **A**) pairs. **(D)** Total time singing by each male during first and third day interaction sessions. Dotted line indicates equal duration of singing on both days. Most males sang more during first day of interaction with a female. The valence of each male during the day 3 session is indicated by a gray scale map, with -1 to 1 indicated by black to white. The amount of singing in the third session was negatively related to valence. **(E)** Fighting during male singing bouts in the first session was associated with low valence during the third session. Typical aggression features associated with low, medium, and higher valence indicated by black, medium gray, and light gray were female retreat, female attack, and male attack/female nonretreat, respectively. Valence scores were significantly lower during day 3 in pairs that fought in day 1 (∗).

To systematically characterize properties of these features across all pairs without knowledge of their relative importance, we performed a PCA. With the first two components, that included 89% of the variance, all pairs were well separated (Figure 2A). From this plot, it is clear that the two position features most strongly distinguishing between pairs are the amount of time spent on the same (X2) and opposite (X5) perches. Further, the distance between birds when on the same perch influences PC1 but not PC2 (Figures 2A,B). The PC1 component thus is positively related to the amount of time birds spent on the same perch, and negatively related to the amount of time on opposite perches, as well as the inter-bird distance while on a perch. This component could discriminate between pairs with varying strengths of affiliation. In contrast, the PC2 component is mainly related to the amount of time birds spent on any perch, whether together or separately. The significance of this component in potentially discriminating pairs is somewhat subtle.

In our system, typical behavior of birds when apparently content (based on posture, and other observations) consisted mainly of sitting on perches. Thus, PC2 may be somewhat independent of birds' ongoing affiliative behaviors, but correlated with an affective state such as low arousal/reactivity or lack of stress. We suspected that this component could influence affiliation status, by distinguishing between pairs based on their potential for successful participation in social interactions.

In order to generate a scalar estimate of pair valence (relative affiliation status, ranging from −1 to +1) we wished to combine these two PC values together. When pairs were separated by combining the values of PC1 and PC2 directly (i.e., for each pair the PC1 and PC2 values from Figure 2A were added), the distribution of valence estimates was highly skewed, with almost half of the values from −0.8 to −1. Most pairs within the negative cluster did not display aggression during this session, and several occasionally displayed positive social behavior such as preening. The inaccuracy of this estimate of pair valence appeared to result from the high variability of PC1 values compared to PC2, so that information contained in the PC2 component was obscured. We thus tested whether more equal weighting of the contributions of PC1 and PC2 could yield a better correspondence with affiliation status. Based on the 4-fold higher variation within the PC1 distribution, we combined the values of PC1∗0.25 + PC2. The resulting valence estimates did correspond well with independently observed social behaviors—pairs with the lowest valences exhibited aggression, and pairs with the highest valences preening.

As an example of characteristic movement and position features of positive and negative valence pairs, perching behavior is shown in Figure 2C. For the upper positive valence pair, the high density of magenta indicates frequent sitting on the same perch. For the negative valence pair, such perch sharing was rare, with more common opposite perch sitting and high rates of moving to and from perches.

In many songbirds, including zebra finches, one male cue on which females could base their judgment is singing frequency or duration (Houtman, 1992; Collins et al., 1994). However, there was no clear relationship between the total duration of singing on day 1 and valence score on day 3 (*r* = −0.33; *p* = 0.18), and the amount of singing on day 3 was negatively correlated with valence score (Figure 2D, *r* = −0.49, *p* = 0.04), which could reflect a reduced need for active courtship in positive valence pairs. Further, most males sang less on day 3 than day 1, consistent with reduced courtship motivation due to beginning of successful or unsuccessful pair bonding (Figure 2D; paired *t*-test, *p* < 0.001).

Because male singing is critical as the initial step in courtship, we further quantified pair behavior during singing periods within the first day's interaction session. During a typical courtship sequence, the male sings to a female, and she either accepts by adopting a CSD posture, or rejects by not. If the male chooses to attempt copulation based on this signal, the female can then continue with copulation or reject him. Surprisingly, there was no clear relationship between the amount of male copulation attempts or successful copulations during singing on day 1, and later valence of pairs. Pairs in which males did or did not attempt copulation during day 1 courtship had similar valence scores on day 3 (*p* = 0.08, *t*-test; copulation attempts valence = 0.18, no attempt valence = −0.20). Further, the success rate (% of completed copulations) of males that attempted copulation was not related to later valence score (*r* = 0.43, *p* = 0.24). Thus, we closely examined behavior of both females and males during bouts of male singing, to identify interaction features related to later valence. The clearest distinction between pairs of birds that had relatively high and low valence during day 3 interactions was aggressive behavior during courtship in the first interaction session. During singing bouts, aggression consisted of beak pecking by either bird, which was responded to with either beak pecking or withdrawal. Pairs that fought during male singing on day 1 had significantly lower valence on day 3 than pairs that did not fight (Figure 2E, *p* = 0.025, *t*-test; aggressive mean = −0.2, *n* = 10; nonaggressive mean = 0.24, *n* = 8). Such fighting was specifically related to courtship singing of males, as it occurred about 50 times more frequently during singing than non-singing periods (across all pairs, the rate of fights/second during singing compared to non-singing median = 54, range 23–162, *p* < 0.01, sign test).

Among the pairs that fought during male singing, we examined whether later valence level was associated with particular interaction features. Pairs that developed the lowest valence tended to have frequent retreats by the female when males attacked during courtship (>50% female retreats, Figure 2E, black). Pairs in which females mainly attacked the male when he sang were associated with somewhat higher valence (≥50% female attacks; Figure 2E, medium gray), while those in which females did not retreat from male attacks were associated with neutral valence levels (>50% male attack, <50% female retreat; Figure 2E, light gray).

### Anatomical analysis

**Figure 3:**
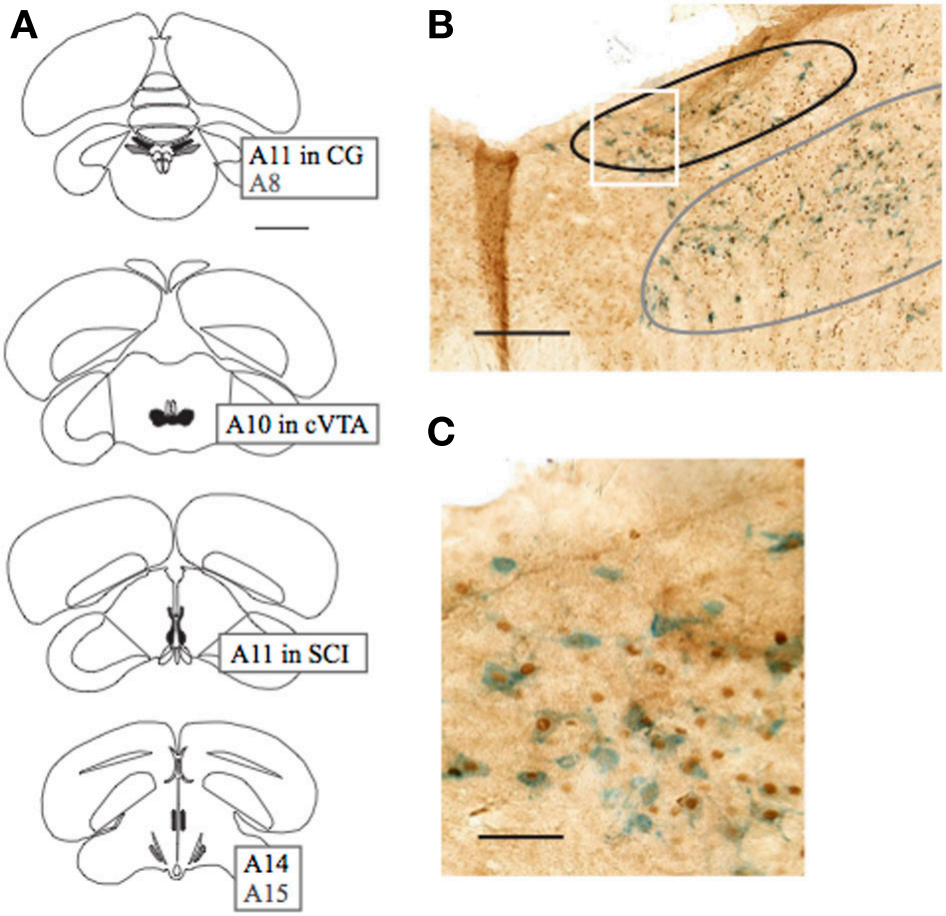
**Schematic diagram of dopaminergic areas in which expression of c-Fos was quantified, and example of colocalization. (A)** From top (caudal) to bottom (rostral), sections included A11 in CG (black) and A8 (gray), A10 in VTA, A11 in SCI, and A14 (black) and A15 (gray). Expression in A8 was not quantified, but is included for reference to **(B)**. **(B)** Image of coronal section containing A11 in CG (black) and A8 (gray) Expression in A8 was not quantified due to difficulty in consistently defining its border. **(C)** Region outlined in white box in **(B)** is expanded to illustrate labeling of dopaminergic neurons with tyrosine hydroxylase (blue) and nuclear expression of c-Fos protein (brown). Scale bars in **(A–C)** indicate 2 mm, 250 μm, and 50 μm, respectively.

Following the third interaction session, which was the basis for calculating valence scores of each pair, the level of neural activation was quantified by immunostaining for the IEG c-Fos protein. Based on results of previous studies (Charlier et al., 2005; Bharati and Goodson, 2006), we quantified expression of c-Fos in putative DA neurons (as defined by expression of Tyrosine Hydroxylase) in five dopaminergic cell groups of the diencephalon and midbrain (Figure 3). In VTA, the one area with boundaries that could be reliably distinguished, expression of c-Fos in non-DA neurons was also quantified.

**Figure 4:**
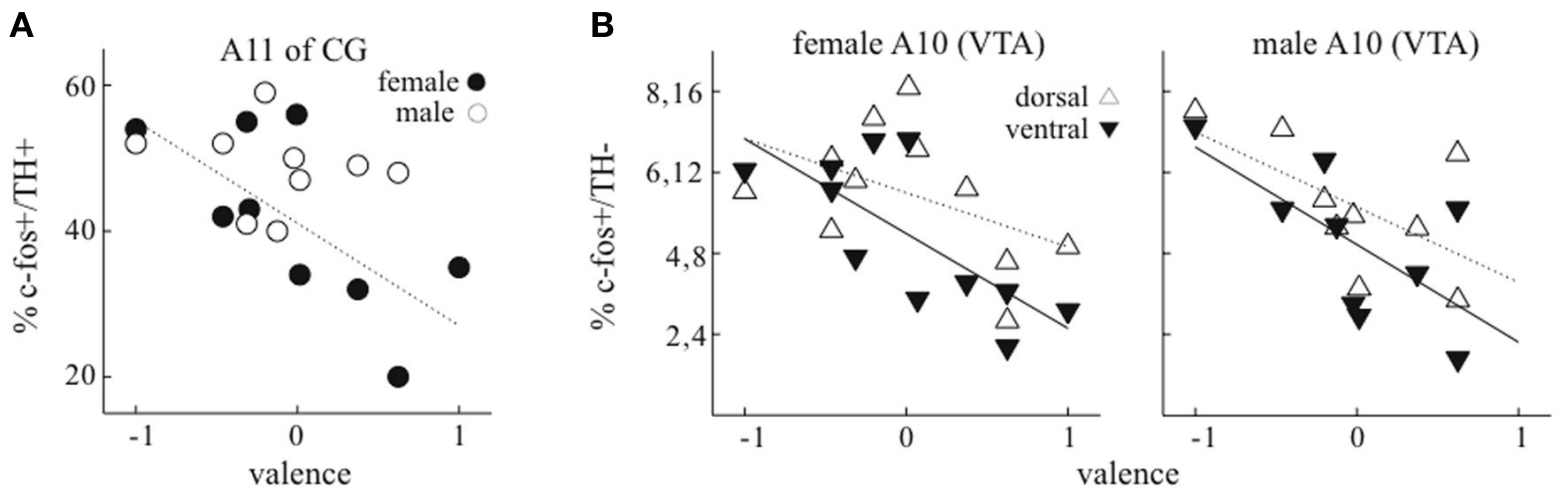
**Relationship of pair valence to expression of c-Fos in dopaminergic and non-dopaminergic neurons. (A)** In dopaminergic neurons in central gray (CG/PAG), the level of expression of c-Fos was negatively correlated with valence in females (filled symbols) but not males (empty symbols). Dotted lines represent linear fit to female data. **(B)** In non-dopaminergic neurons in VTA, for females there was a stronger negative relationship of valence to c-Fos expression in ventral than dorsal VTA, and for males this negative relationship was similar in both regions. Because the level of expression in dorsal VTA was about double that in ventral VTA, the scale of y-axis indicates separate percentages for both ventral (lower values) and dorsal (higher values) regions.

The level of c-Fos expression was clearly related to pair valence scores in putative DA neurons of A11 of CG and in non-DA neurons of VTA. For A11 (CG), the level of c-Fos expression in dopaminergic neurons was much lower in females with positive valence (Figure 4A, *r* = −0.6861, *p* = 0.041, *n* = 9). In contrast, expression in males was similar for all valence levels (*p* = 0.77, *n* = 9). In DA neurons of other areas, there was no clear relationship between the level of c-Fos expression and valence scores (Supplementary Figure 1).

In VTA, the level of c-Fos expression in non-DA neurons was also related to pair valence, and dependent on the sex of birds. As recent studies in mammals indicate that dorsal and ventral regions of VTA can have distinct functions (Lammel et al., 2008; Brischoux et al., 2009), we separately quantified the level of c-Fos expression in these regions. In both females and males, about double the number of non-DA neurons expressed c-Fos in the dorsal compared to the ventral VTA. In females, the level of expression in ventral VTA was negatively correlated with valence scores (Figure 4B; *r* = −0.62, *p* = 0.04), while a similar trend in dorsal VTA was not significant (*r* = −0.44, *p* = 0.18). In males there was a similar tendency of negative relationship of c-Fos expression to valence score in both dorsal and ventral VTA (ventral: *r* = −0.68, *p* = 0.045; dorsal: *r* = −0.66, *p* = 0.05).

## Discussion

With a simple video tracking system, we could automatically quantify affiliative behavior of pairs of female and male finches. Across all pairs, there was a range of affiliation, from strong (positive valence) to weak (negative valence). In two dopaminergic areas, the level of neural activation was related to pair valence. In A11 (in CG) of females, but not males, expression of the IEG product c-Fos in putative DA neurons was selectively reduced in females within positive valence pairs, while in VTA there was a negative correlation between pair valence and activation of non-DA neurons, which was stronger in dorsal than ventral regions for females. These results suggest that the intense social interactions of courtship and pair formation involve regulation of negative emotional systems in the brain, rather than only positive ones.

### Anatomical results

In both A11 in CG and non-DA neurons in VTA, the level of expression of c-Fos had a specific relationship to the valence of pairs, and was dependent on sex. These two dopaminergic areas adjoin each other in birds, with A11 located within the central gray (CG, overlapping with periaqueductal gray (Appeltants et al., 2000; Kingsbury et al., 2011 also referred to as A10dc, Zahm et al., 2011). Song system nuclei of males receive input from dopaminergic neurons in both areas, with the striatopallidal Area X only from A10 of VTA (Lewis et al., 1981) and the motor control nuclei HVC and RA from both A11 of CG and A10 of VTA (Appeltants et al., 2000, 2002). It is likely that more general interaction of these areas with motivational and affective systems plays an important role in both sexes. In birds, dopaminergic neurons in both A11 of CG and A10 of VTA receive input from the medial preoptic nucleus of the hypothalamus (mPOA), which regulates sexual motivation (Berk and Butler, 1981; Balthazart and Absil, 1997; Riters and Alger, 2004). Beyond these specific afferent and efferent connections, quite extensive projections of VTA to avian forebrain areas have been demonstrated (Bottjer, 1993). In mammals, the dopaminergic A11 group (in PAG, homologous to avian CG) has many similar projection targets as A10 of VTA, but some unique ones such as the central nucleus of the amygdala and bed nucleus of the stria terminalis (Hasue and Shammah-Lagnado, 2002).

Recent studies highlight some important role of the dopaminergic A11 group in social behavior of birds. These neurons are more strongly activated in males that engaged in either courtship or sexual behavior with females than in non-interacting males (Charlier et al., 2005; Bharati and Goodson, 2006). While this distinction may indicate a specific function in these intense social behaviors, it could also reflect a higher arousal of courting compared to non-social males. Studies that reported a correlation of singing activity with A11 dopaminergic activation (Lynch et al., 2008; Goodson et al., 2009) may also reflect variability in baseline arousal level of males. Further, the level of c-Fos expression in a previous study (~50%) related to singing or sexual behavior was similar to that of all males in this study, and of females in negative valence pairs. In contrast, females in positive valence pairs had similar levels of expression in A11 of CG neurons as non-courting males of earlier studies (Maney and Ball, 2003; Goodson et al., 2009). Thus, this suppression of A11 of CG function could reflect a reduction of females' arousal related to acceptance of a male's presence.

Both dopaminergic and non-dopaminergic neurons in VTA have been shown by a variety of methods to be involved in songbird courtship (Yanagihara and Hessler, 2006; Hara et al., 2007; Huang and Hessler, 2008; Lynch et al., 2008; Goodson et al., 2009). During courtship singing compared to non-courtship singing, the firing rate of presumed DA neurons was higher, while that of presumed non-DA neurons could either increase or decrease (Yanagihara and Hessler, 2006). Further, the strength of synapses onto DA but not non-DA neurons in VTA was increased following courtship singing but not singing while alone (Huang and Hessler, 2008). In mammals, a similar activation of DA neurons is associated with artificial and naturally occurring rewards such as drugs and food (Stuber et al., 2008). In studies such as this one examining IEG expression in VTA, non-DA neurons are typically more strongly activated during courtship interactions than DA neurons (Hara et al., 2007; Lynch et al., 2008; Goodson et al., 2009). However, this distinction may be due to a relatively low level of IEG expression in DA neurons. In parallel with these studies characterizing VTA activity, a variety of experiments have demonstrated that directed courtship singing is associated with increased dopamine release in a major target of VTA, Area X (Sasaki et al., 2006; Leblois et al., 2010). Based on these previous studies, the activity level of both non-DA and DA neurons in VTA appear similarly dependent on intense social behaviors like courtship. While this may appear contradictory, given the inhibition of DA by non-DA neurons, a consideration of phasic activation patterns of both could suggest a mechanism. Recent studies suggest that phasic burst firing of DA neurons is critically dependent on phasic removal of inhibitory input, including that from local non-DA interneurons (Lobb et al., 2010, 2011).

Based on this model, there was a negative correlation between the level of activation of non-DA and DA neurons in ventral VTA of females and pair valence scores. Females with strong neural activation in ventral VTA tended to have low valence scores. Although similar studies have not yet been done in birds, in mammals there are clear distinctions between dopaminergic neurons in ventral and dorsal VTA in anatomical projection targets, physiological properties, and responses to rewarding vs. aversive stimuli (Lammel et al., 2008; Brischoux et al., 2009). While activity of DA neurons in dorsal VTA is associated with positive events, activity of those in ventral VTA was associated with negative events such as electric shocks (Brischoux et al., 2009; Matsumoto and Hikosaka, 2009; Lammel et al., 2011). Thus, stronger neural activation in ventral VTA of females in low valence pairs could reflect the negative social experience of interacting with a non-preferred male. The similar relationship of valence to neural activation of dorsal and ventral VTA in males may reflect a reduction of both withdrawal and approach motivation during an early stage of pair formation. While there was no clear relationship of valence to dopaminergic activation in VTA, unlike in a previous study examining various social behaviors (Goodson et al., 2009), this may reflect a low level of activity-dependent c-Fos expression in this area.

### Behavioral results

In complex interactions such as courtship, it is likely that full and normal activation of critical neural systems will only occur when both individuals can freely choose how to interact with the other. Here, we attempted to provide such opportunities by allowing females and males free movement within a cage. In pairs that had begun to initiate pair bond formation, as shown by the presence of close physical contact, movement was generally less agitated than in lower valence pairs (e.g., Figure 1B). This behavioral characteristic was associated in females, but not males, with reduced activation of A11 of CG neurons, as discussed above. Such a distinction in neural activation between females and males may reflect a higher level of judgment by females, in contrast to more indiscriminate mating motivation of males. Further quantification of each individual's relative affiliation status, rather than the average within pairs, would be useful in cases of divergent motivation.

It has been proposed that during courtship, both birds must balance opposing tendencies to approach and flee from the other bird (Hinde, 1953). The negative affective tendency may reflect some general fear of close physical contact, as this often occurs during negative experiences such as aggression or predation (Porges, 2003). Thus, development of behavioral increase in affiliation may be associated with or require suppression of negative emotional affect and responsiveness, rather than only an increase of positive affect.

Here we examined directly how behavioral interactions between female and male during the male's initial courtship bout influenced eventual pair valence, or level of affiliation. Male courtship in later low valence pairs was often associated with aggression. While such aggression by the female bird toward the male when he sings seems to indicate a strong negative response to his courtship, it was somewhat surprising that males also sometimes attacked the female while they sang. However, in territorial songbirds, singing is used in both a “sexual” context to attract a female bird and an “aggressive” context to repel competitor males. Further, domesticated male zebra finches occasionally sing to other males in an apparently aggressive context (Hessler and Doupe, 1999). In general, previous experimental and observational studies suggest that highly aroused sexual and aggressive behavior may have some common neural mechanisms (Veening et al., 2005).

Such occurrence of aggressive behavior during courtship interactions may indicate that females' judgment of males is based on more than just song features. Specifically related to courtship singing bouts, a male's responses to feedback given by the female can influence her decision to mate or not. Male cowbirds and bowerbirds modulate the intensity of their courtship to avoid startling their target female (Patricelli et al., 2002; O'Loghlen and Rothstein, 2012), and male cowbirds can modify their singing output to feature elements to which females respond positively (West and King, 1988).

Recent advances in computational efficiency that allow automated monitoring of animal behavior allow more efficient and comprehensive analysis critical for ethological studies, and especially useful for characterizing interaction of multiple individuals (Branson et al., 2009; Dankert et al., 2009). Further development of our video tracking system will aim to automatically identify specific common behaviors, such as eating and grooming. Such information, as well as more detailed quantification of individual movement patterns, should allow more accurate assessments of each individual's judgment of their partner. Of more general utility, this system can be used for tracking other small animals moving in three-dimensional space, such as fish, and its implementation in the commonly available program Matlab allows easy customization by users.

This study, by assaying IEG expression, could quantify the level of neural activation within an entire interaction session. It will also be interesting to test whether neural activity in A11 of CG and VTA (both A10 dopaminergic and non-dopaminergic neurons) is acutely related to ongoing positive or negative interactions. As in previous studies focusing only on males (Yanagihara and Hessler, 2006), this could be done with acute single-unit recordings in semi-restricted birds, though it will be especially useful to record simultaneously from both birds in a setting where they can freely choose whether to interact or not. Clearly, the complex dyadic interaction during courtship involves additional neural systems besides the dopaminergic one examined here. Further studies using similar behavioral tests should also characterize peptidergic systems such as vasopressin/oxytocin that control the development of close social bonds in birds and mammals (Goodson and Thompson, 2010; Insel, 2010; McCall and Singer, 2012).

### Conflict of interest statement

The authors declare that the research was conducted in the absence of any commercial or financial relationships that could be construed as a potential conflict of interest.

## Supplementary material

Supplementary Figure 1 **Lack of relationship between pair valence and c-Fos expression in additional dopaminergic areas**. In A11 of SCI, A14, A15, and A10 of caudal VTA (cVTA), the percentage of dopaminergic neurons expressing c-Fos was not significantly related to valence of females (filled symbols) or males (empty symbols).
