# Supplementary figures and images for: Sexually dimorphic activation of dopaminergic areas depends on affiliation during courtship and pair formation
Source: Front Behav Neurosci. 2014 Jun 11;8:210. doi: 10.3389/fnbeh.2014.00210 (PMC4052804; doi:10.3389/fnbeh.2014.00210)

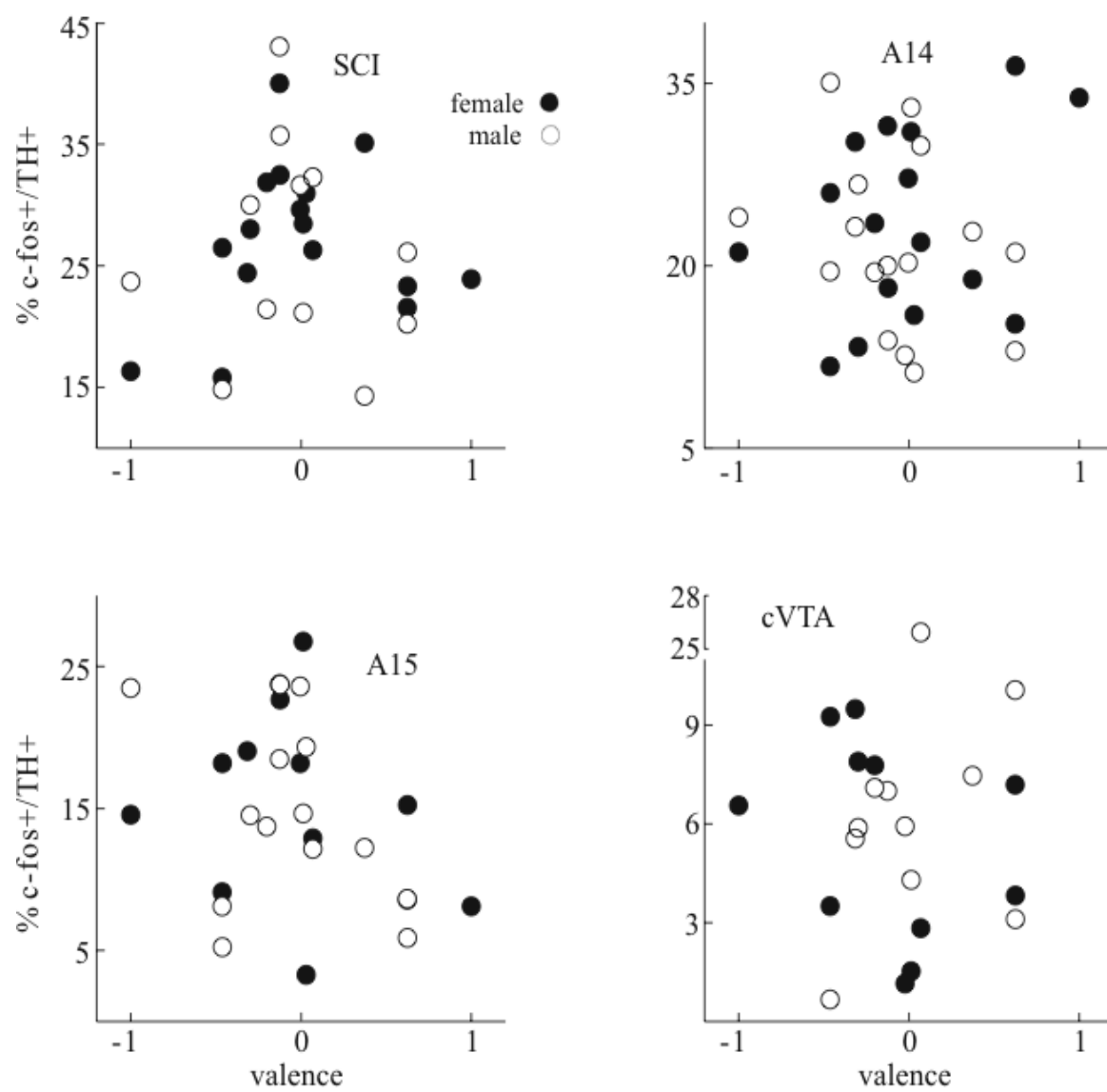

Supp. Fig. 1

Supplement: Supplementary Figure 1 — Lack of relationship between pair valence and c-Fos expression in additional dopaminergic areas. In A11 of SCI, A14, A15, and A10 of caudal VTA (cVTA), the percentage of dopaminergic neurons expressing c-Fos was not significantly related to valence of females (filled symbols) or males (empty symbols). [file Presentation1.PDF]
